# Altered Transcriptional Control Networks with Trans-Differentiation of Isogenic Mutant-KRas NSCLC Models

**DOI:** 10.3389/fonc.2014.00344

**Published:** 2014-12-08

**Authors:** John A. Haley, Elizabeth Haughney, Erica Ullman, James Bean, John D. Haley, Marc Y. Fink

**Affiliations:** ^1^Department of Biomedical Sciences, LIU Post, Brookville, NY, USA; ^2^Regeneron Pharmaceuticals Inc., Tarrytown, NY, USA; ^3^Infectious Disease Division, Memorial Sloan Kettering Cancer Center, New York, NY, USA; ^4^Department of Pathology, Cancer Center, Stony Brook School of Medicine, Stony Brook, NY, USA

**Keywords:** tumor heterogeneity, epigenetic, transcription, systems biology, EMT

## Abstract

**Background:** The capacity of cancer cells to undergo epithelial mesenchymal trans-differentiation has been implicated as a factor driving metastasis, through the acquisition of enhanced migratory/invasive cell programs and the engagement of anti-apoptotic mechanisms promoting drug and radiation resistance. Our aim was to define molecular signaling changes associated with mesenchymal trans-differentiation in two KRas mutant NSCLC models. We focused on central transcription and epigenetic regulators predicted to be important for mesenchymal cell survival.

**Experimental design:** We have modeled trans-differentiation and cancer stemness in inducible isogenic mutant-KRas H358 and A549 non-small cell lung cell backgrounds. As expected, our models show mesenchymal-like tumor cells acquire novel mechanisms of cellular signaling not apparent in their epithelial counterparts. We employed large-scale quantitative phosphoproteomic, proteomic, protein–protein interaction, RNA-Seq, and network function prediction approaches to dissect the molecular events associated with the establishment and maintenance of the mesenchymal state.

**Results:** Gene-set enrichment and pathway prediction indicated BMI1, KDM5B, RUNX2, MYC/MAX, NFκB, LEF1, and HIF1 target networks were significantly enriched in the trans-differentiation of H358 and A549 NSCLC models. Physical overlaps between multiple networks implicate NR4A1 as an overlapping control between TCF and NFκB pathways. Enrichment correlations also indicated marked decrease in cell cycling, which occurred early in the EMT process. RNA abundance time course studies also indicated early expression of epigenetic and chromatin regulators within 8–24 h, including CITED4, RUNX3, CMBX1, and SIRT4.

**Conclusion:** Multiple transcription and epigenetic pathways where altered between epithelial and mesenchymal tumor cell states, notably the polycomb repressive complex-1, HP1γ, and BAF/Swi-Snf. Network analysis suggests redundancy in the activation and inhibition of pathway regulators, notably factors controlling epithelial cell state. Through large-scale transcriptional and epigenetic cell reprograming, mesenchymal trans-differentiation can promote diversification of signaling networks potentially important in resistance to cancer therapies.

## Introduction

Cellular plasticity in epithelial cancers is associated with a progression to a metastatic state (1, 2) and resistance to anti-cancer therapies (3, 4). Over 90% of cancer patient deaths are attributable to complications arising from metastatic dissemination of cancer cells to distant organ sites. Tumor plasticity associated with epithelial mesenchymal transition (EMT) (5, 6) contributes to metastasis, drug resistance and is correlated with poor prognosis (7, 8). The epigenetic reprograming associated with EMT promotes the disassembly of epithelial cell-junctions, a loss of epithelial polarity (4, 9), and the formation of molecular assemblies allowing cell migration and invasion (10). In multiple model systems, EMT-derived mesenchymal cells can show the properties of cancer stem cells including tumor initiation at low cell inoculation *in vivo*, sphere formation *in vitro*, and CD44^high^, CD24^low^, and ALDH1^active^ pluripotent stem cell markers (11, 12). Despite advances in the treatment of non-small cell lung cancers (NSCLC), for example, the development of EGFR tyrosine kinase inhibitors (EGFR TKIs) for patients with activating EGFR mutations, survival rates for patients with mutant-KRas lung cancer are poor. Mutant-KRas is observed in ~20% of NSCLC cases and is generally independent of EGFR mutations (13). In a recent clinical study, the median survival time of patients treated with standard of care was 7.7 months in NSCLC patients with mutant-KRas, in marked contrast to 38 months in patients with mutant EGFR (14). Several studies suggest tumor cells harboring mutant-KRas may be primed to undergo epithelial mesenchymal trans-differentiation, for example (15, 16). These mesenchymal-like carcinoma cells have been shown to be resistant to many conventional lung cancer therapies, including taxanes, pemetrexed, gemcitabine, and EGFR TKIs (8, 12, 17). Chemotherapy has been shown to promote selection for EMT-derived cells in a number of solid tumor types (7, 17, 18) and conversely markers of EMT appears to contribute to chemotherapy resistance (19) and predict response to EGFR and PI3K inhibitors (20). Similar data show EMT-derived cells can serve as reservoirs for cancer recurrence in relevant genetically engineered models (21, 22). Therapies are needed that specifically target drug resistant mesenchymal-like tumor cells. However, survival signaling networks in EMT–derived cells appear heterogeneous and critical dependencies common to mesenchymal tumor cells remain ill defined. Current inhibitors of mesenchymal stem-like tumor cells have been largely restricted to specific tumor and driver types, for example, JAK or PDGFR inhibitors. Since mesenchymal trans-differentiation involves an epigenetic reprograming, the pharmacological use of epigenetic modulators would appear attractive. However, initial therapeutic successes with single agent DNA methyltransferase inhibitors, HDAC inhibitors, and EZH2 inhibitors have been more pronounced in the hematologic malignancies, as opposed to epithelial-derived carcinomas, which can undergo EMT.

In order to understand and identify transcriptional and epigenetic networks in a mutant-KRas mesenchymal cell context, we have modeled metastable reversible EMT in two NSCLC cell backgrounds. “Metastable” refers to a reversible EMT, achieved for example by withdrawal of inducer (23), where in intermediate stages cells can express both epithelial and mesenchymal markers (24). Here, we compared CD44^low^ A549 cells with isogenic CD44^high^ A549/transforming growth factor beta (TGFβ) cells and CDH1/E-cadherin^high^ H358 cells with isogenic CDH1^low^ H358/dox-TGFβ cells. As expected both models reversibly undergo EMT and are less responsive to paclitaxel, gemcitabine, and erlotinib in the mesenchymal state (data not shown). These models confirm the clinical observations and show that tumor cells that have undergone EMT show increased resistance to standard of care cancer treatments. Importantly, upon undergoing EMT tumor cells acquire novel mechanisms of cellular signaling and resistance to apoptosis not apparent in their epithelial counterparts.

We sought to define at signaling network changes distinguishing epithelial and mesenchymal tumor states, with an aim of identifying key nodes important in mesenchymal cell survival. Pharmacological blockade of mesenchymal survival pathways may overcome a limitation of current therapies, which preferentially impact tumor cells with an epithelial phenotype (17). We used a combination of RNA-Seq, phosphoproteomic, and bioinformatics approaches to identify transcriptional and epigenetic networks modulated as a consequence of epithelial mesenchymal transition (EMT). We employed TGFβ to induce EMT in the A549 and H358 NSCLC models, where cells were preselected for a starting epithelial state. TGFβ is a physiologically relevant inducer of EMT (25) associated with a macrophage/monocyte rich inflammatory microenvironment (26). Detailed RNA, protein, and phosphopeptide abundance datasets were obtained. Using pathway prediction and gene-set enrichment approaches, and protein–protein interaction data we observed altered regulation of transcriptional, epigenetic, and chromatin modulators and explored intersections between key pathways.

## Materials and Methods

### Cell culture conditions

Parental H358 and A549 cells were obtained from the ATCC and maintained in RPMI 1640 containing 10% FCS. Both H358 and A549 are related adenocarcinoma NSCLC lines, where both express an activated KRas oncogene, with mutations at G12C and G12S, respectively. H358 cells harbor pathogenic mutations in CTNNB1, KRAS, LIMK1, MAX, MED12, MSH3, PML, RNF212, RUNX2, SATB2, SF3B1, SGK3, TP63, and USP2. A549 adenocarcinoma NSCLC cells harbor pathogenic mutations in DCLK3, FRMD6, KEAP1, KRAS, PTPN21, and TCEA2 (27). Both cell lines undergo EMT in response to TGFβ. All cells were kept at 37°C with 5% CO_2_. The TGFβ inducible variant of H358 and its dox-vector control line were both engineered from a single H358 cell clone with marker expression CDH1^high^ and epithelial cell-junctions. Multiple H358-dox-TGFβ cell lines were isolated (23, 24) using the doxycycline inducible CMV promoter (pTRE2puro; invitrogen.com) using the two vector TET repressor/TET activator system (ptTS, prTA; blaR) based on Ref. (28). H358-dox-TGFβ clones were maintained in RPMI 1640, 10% tetracycline-free FCS (clontech.com), L-glutamine (1 mM), sodium pyruvate (1 mM), D-glucose (4.5 g/l), 10 mM HEPES, blasticidin (10 μg/ml), and doxycycline (0.5 μg/ml). Doxycycline (0.5 μg/ml) induction of transgene expression was verified by immunoblot and was shown to correctly modulate EMT marker expression (Figure S1 in Supplementary Material). Inducible RNA expression (log2 FC = 4.6) is shown in Table S1 in Supplementary Material. Parental A549 cells were anti-CD44 antibody selected using magnetic beads into a CD44^low^ starting epithelial population, which was subsequently induced with TGFβ (10 ng/ml) to yield a uniformly mesenchymal cell population after 7 days.

### RNA-Seq

Duplicate cell samples (~3 × 10^6^ cells) were lyzed in 600 μl Qiagen RLT buffer, 1% β-mercaptoethanol, followed by column isolation (qiagen.com). RNA isolation, library construction, and RNA-Seq essentially followed Illumina protocols (illumina.com). cDNA was prepared from total RNA with globin RNA reduction, followed by library generation (Illumina True Seq). cDNA libraries were immobilized (Illumina, Standard Cluster Generation Kit) and sequenced on an Illumina HySeq instrument. Read depths for H358 and A549 were 30 million and 25 million, respectively. RNA-Seq was performed on Illumina HySeq instrument^1^. FASTQ file reads passing Illumina purity filter (PF reads) were aligned and quantitation performed by RSEM (29), generating files where normalized counts for each detected gene and isoform (UCSC Known Gene). Aligned RNAs (27976 genes; 48241 isoforms) passing QC thresholds were used to calculate mesenchymal–epithelial transcript abundance ratios followed by log2 linear scaling. Additional analysis was performed using Cufflinks (30) on BAM files aligned to hg19. For RNA-Seq data were selected where log2 fold change (FC) was ≥1.0 and where rsem reads were >5 in any sample. In difference analysis where either numerators or denominators were equal to zero, values were generated (specified as >5 or <5) in gene lists if differences >10 rsem reads were observed in both H358 and A549 models with concordant log2 signs. Concordance required >2 FC in three out of four samples (Mes-1 and Mes-2 over Epi-ave) for both A549 and H358 cell models.

### Preparation of cell extracts and phosphorylation directed affinity chromatography

Essentially, site-specific serine and threonine phosphorylation changes were measured by SILAC labeling of proteins, trypsin digestion, ion-exchange fractionation, and TiO2 affinity selection in the presence of 1 M lactic acid to minimize acidic peptide binding followed by nano LC-MS/MS. Site-specific tyrosine phosphorylation was measured by SILAC labeling, peptide anti-phosphotyrosine selection, and nano LC-MS/MS. Total protein changes were assessed by SILAC labeling, ion-exchange fractionation, and LC-MS/MS.

Approximately 5 × 10^8^ H358 or A549 mesenchymal and epithelial cells were cultured in RPMI 1640 media containing heavy (^13^C6) arginine and lysine or light (^12^C) arginine and lysine, respectively [SILAC; (31)]. Cells from five biological replicates were extracted in 8 M urea, 50 mM HEPES, 2.5 mM sodium pyrophosphate, 1 mM β-glycerophosphate, 1 mM sodium orthovanadate, and phosphatase inhibitor cocktails 1 and 2 (1:100 dilution; sigmaaldrich.com). Heavy and light extracts were mixed, sonicated for 2 × 30 s and centrifuged at 10,000 × *g*. The combined supernatant was subjected to reduction (5 mM tributyl phosphine), alkylation (15 mM iodoacetamide), proteolysis with trypsin, desalting on C18 resin (Sep-Pak C18; waters.com) and lyophilization. Peptides were immunoprecipitated with anti-phosphotyrosine affinity resin essentially as described (32), with C18 cartridge (Opti-Lynx 40 μg C18AQ; optimizetech.com) desalting with a 10 min gradient elution with inline UV absorbance detection at 220 nm using a 250 nl flow cell. Peptides were lyophilized, resuspended in 0.1% formic acid, and analyzed by LC-MS/MS.

Anti-phosphotyrosine flow through peptides were separated by SCX ion-exchange chromatography (SampliQ resin 150 mg; agilent.com) in 10 mM KH_2_PO_4_ pH 3.0, 25% acetonitrile step eluted with increasing KCl (20, 40, 60, 80, 100, 125, 150, 200, 350, 500 mM KCl) yielding ten fractions. Samples were desalted by C18 step chromatography (OASIS 60mg C18; waters.com), followed by TiO_2_ affinity chromatography (10 u beads; glsciences.com) using 3 mg TiO_2_ beads per fraction in 100 ul 0.1% TFA, 50% acetonitrile (ACN), where 1 M lactic acid was used to suppress non-specific binding. Phosphopeptides were eluted with 50 mM KH_2_PO_4_ pH 10.5 (pH adjusted with NH_4_OH), immediately neutralized with 5% formic acid, 50% acetonitrile, and lyophilized. Peptide fractions were resuspended in 0.1% FA and analyzed by LC-MS/MS. Peptides not binding to TiO_2_ (flow through fraction) were desalted (OASIS 60 mg C18; waters.com), lyophilized and protein concentration determined by micro BCA assay (piercenet.com). Peptide fractions were resuspended in 2% ACN, 0.1% FA, and analyzed by LC-MS/MS.

### Peptide identification and quantification by LC-tandem MS

LC-MS/MS was performed essentially as previously described (33, 34). Two (A549) or three (H358) LC-MS/MS experiments were performed for each fraction (1 fraction for pY, 10 fractions for SCX/TiO2, and 10 fractions for SCX/total peptide). Proteins were identified from survey and product ion spectra data, using the Paragon algorithm of ProteinPilot [v4.0; (35)] and GPM [(36) v2.2.1]. Two missed tryptic cleavages were allowed and posttranslational modifications considered included cysteine derivitization, STY phosphorylation, deamidation, carbamylation, oxidation, and SILAC labels. Database searches used the human UniProt FASTA database (10-2012; 70,391 sequences including common contaminants). When multiple protein isoforms were identified, Protein Pilot allowed only peptides specific to each detected isoform to be used, which factored in ion counts for weighting in the protein ratio calculation (37). Parsimony of protein results was assured by rigorous protein inference with the ProGroup algorithm. Protein identification complied with the guidelines of Bradshaw et al. (38) where two or more unique isoform-specific peptides were required for inclusion. False discovery rates of phosphotyrosine peptide capture experiments were <1%. False positive rates for the high complexity TiO2 and total peptide assignments ranged between 1.4 and 0.8%. For statistical analysis we required four or more peptides with individual peptide assignments at >95% confidence. Phosphopeptide peak areas were normally distributed by log10 + 1 conversion followed by paired *t*-test. Phosphopeptides sites identified four or more times are listed in Table S2 in Supplementary Material and coordinate changes in phosphopeptide abundance between H358 and A549 models were observed for 73 unique sites are listed in Table S3 in Supplementary Material. Fifty-eight protein abundance changes were coordinately associated with H358 and A549 trans-differentiation, with two or more unique peptides also at 95% peptide ID confidence are listed in Table S4 in Supplementary Material. Co-correlation of overlapping peptides from skipped cleavage and multiple charge states were used to further reduce false discovery rates and correctly bin previously defined benchmark peptides from E-cadherin, vimentin, and fibronectin to their respective epithelial or mesenchymal cell states.

### Functional analysis and performance parameters

**Figure 1:**
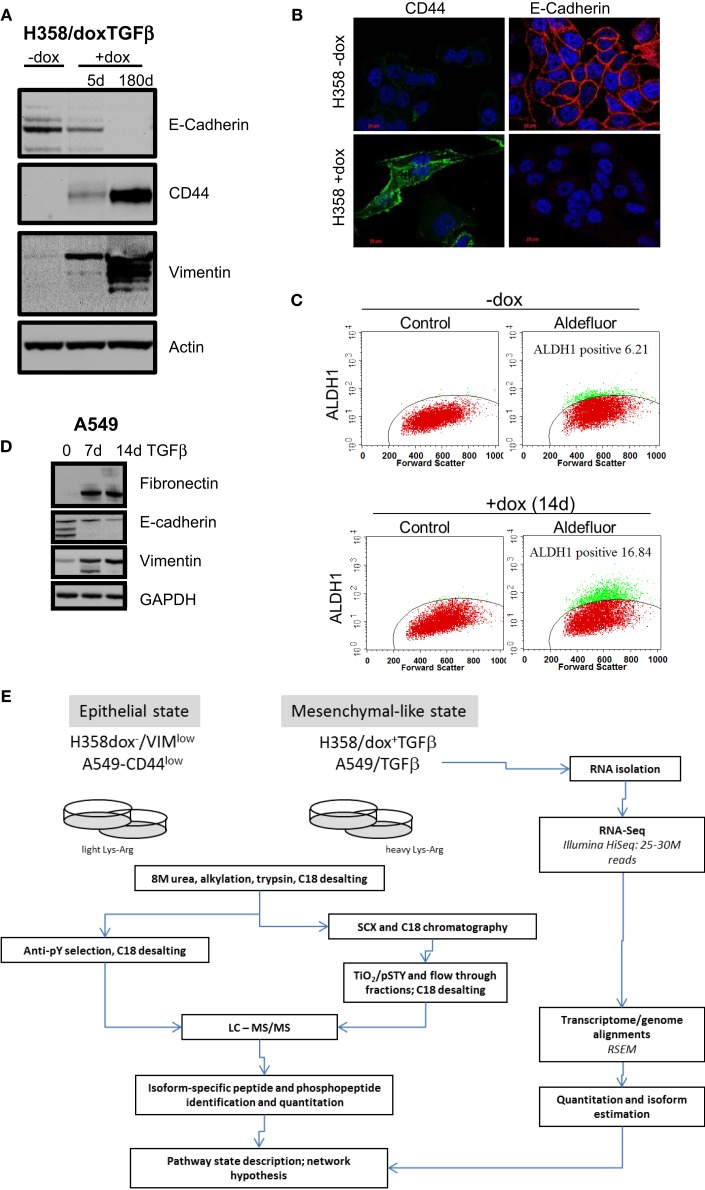
**Marker expression in H358 and A549 isogenic mesenchymal trans-differentiation models**. **(A)** Immunoblot staining for E-cadherin, CD44, vimentin, and actin (control) in the H358-dox-TGFβ model in epithelial (−dox) or mesenchymal (+dox) states (5 days and ~180 days on doxycycline). **(B)** Deconvoluted fluorescence microscopy image for CD44 (green) and E-cadherin (red) in the H358-dox-TGFβ model in epithelial (−dox) or mesenchymal (+dox) states. **(C)** H358 cells induced to express TGFβ for 14 days (bottom panels) show increased aldefluor activity, a marker of aldehyde dehydrogenase activity and stemness, relative to control cells (top panels), as measured by FACS. **(D)** Immunoblot staining for fibronectin, E-cadherin, vimentin, and GAPDH (control) in the A549 cell in the presence or absence of exogenous TGFβ (10 ng/ml) for 7 or 14 days. **(E)** A workflow schema for RNA, protein, phosphopeptide abundance measurement, and cross-correlation.

A schema for the statistical and categorical analysis of protein, phosphopeptide, and RNA transcript data is provided in Figure 1. Changes in the abundance of proteins, phosphopeptides, and RNA transcripts were compared between fractions, experiments, and models where membership within specific signaling networks only was established using two or more independent lines of evidence. Cross-correlation between two independent isogenic KRas models was used to reduce noise and reduce false positives. Proteins, phosphopeptides, and RNA transcripts differentially expressed between EMT-like cell states were grouped by function using literature, gene-set enrichment (GSEA) (v14; broadinstitute.org/gsea), Gene Pattern (v3.8.1; broadinstitute.org/cancer/software/genepattern), DAVID^2^, and Ingenuity pathway prediction (IPA; ingenuity.com). In the functional illustrations of complexes modulated during EMT, proteins were assembled by protein–protein contacts [BioGrid; thebiogrid.org/ (39); STRING; string-db.org] and further grouped manually by biological systems or “machines.” The function of individual peptide phosphosites was evaluated using PhosphoSite [phosphosite.org; (40)] and published literature. Venn overlaps were calculated using (bioinformatics.psb.ugent.be/webtools/Venn/).

### Reporter plasmid transfection

SuperTOP plasmid is a TCF–LEF responsive promoter driving expression of luciferase (addgene.com). The control superFOP contains mutated TCF/LEF promoter binding sites and serves as a negative control. The TK-renilla plasmid serves to normalize for DNA uptake. Plasmids were isolated from DH5a *E. coli* using an endotoxin-free isolation (qiagen.com). H358-dox-TGFβ cells were plated into 12 well plates (~2 × 10^5^ cells/well). Super TOP, super FOP, and control Renilla plasmids (promega.com), were transfected using Lipofectamine 2000 (lifetechnologies.com) and reporter assays conducted as defined by the manufacturer (promega.com). After normalization to Renilla control signal, TOP and FOP data were expressed in relative light units (RLUs).

### Immunblot and immunofluorescence

Gel electrophoresis and immunoblot of H358 and A549 cell extracts were performed under standard conditions using ECL Plus Western Blotting Substrate (piercenet.com) using vimentin (1:5000 dilution; bdbiosciences.com) and E-cadherin antibodies (1:1000 dilution; cellsignal.com). Immunofluorescence was performed under standard conditions using β-catenin (1:100 dilution), E-cadherin (1:200 dilution), and CD44 antibodies (1:400 dilution), all from Cell Signaling Technologies (cellsignal.com) with DAPI staining. Cells were imaged using a Zeiss Axiovert inverted fluorescence microscope at 60× magnification.

## Results

### Characterization and validation of H358 and A549 mutant-KRas models

Two KRas mutant adenocarcinoma NSCLC cell lines H358 and A549 were used as model systems to molecularly define transcriptional and epigenetic reprograming following mesenchymal trans-differentiation. H358 and A549 cells can spontaneously generate populations of CDH1^high^/CD44^low^ and CDH1^low^/CD44^high^, with epithelial and mesenchymal-like phenotypes, respectively (41). Spontaneous inter-conversion has been previously reported (42, 43). H358 contain relatively rare (estimated at ~2–4%) CDH1^low^/CD44^high^ cells while A549 are typically a more mixed population of each phenotype. As such, all H358 experiments were initiated from epithelial CDH1^high^/VIM^low^ clones with predominant epithelial cell-junctions, from which subsequent H358/dox-TGFβ clones were derived. Doxycycline (0.5 μg/ml) induction of transgene expression, a constitutively active form of TGFβ1 (37), was verified by immunoblot and was shown to correctly modulate EMT marker expression (CDH1^low^, CD44^high^, and VIM^high^) as shown in Figure 1A. Fluorescence microscopy (Figure 1B) showed loss of E-cadherin membrane localization and gain of CD44 expression in H358/dox-TGFβ cells relative to the minus dox control. Multiple H358/dox-TGFβ clones exhibited correct isogenic mesenchymal trans-differentiation and in contrast vector control cells remained epithelial in the presence of doxycycline (24). Similarly, the percentage of aldefluor positive cells, a marker of aldehyde dehydrogenase activity and putative stemness, was increased after mesenchymal trans-differentiation with TGFβ for 14 days. The percentage of aldefluor positive cells was 6.2% in the −dox control H358 cells and 16.8% in the +dox H358/TGFβ producing cells (Figure 1C). A549 cells were anti-CD44 antibody counter selected using magnetic beads into a CD44^low^ starting epithelial population, which was subsequently induced with TGFβ (10 ng/ml) to yield a uniformly mesenchymal cell population after 14 days. This allowed a more direct comparison of the CD44^low^ starting population with the TGFβ-induced mesenchymal population, which became CD44^high^. Immunoblot for fibronectin, E-cadherin, and vimentin confirmed an EMT transition after 7 and 14 days exposure to TGFβ in A549 cells (10 ng/ml; Figure 1D). EMT occurs in H358 and A549 epithelial CDH1^+^/CD44^low^ subclones exposed to TGFβ over a prolonged period.

### Integration of RNA, protein, and phosphoprotein EMT state-specific measurements

**Figure 2:**
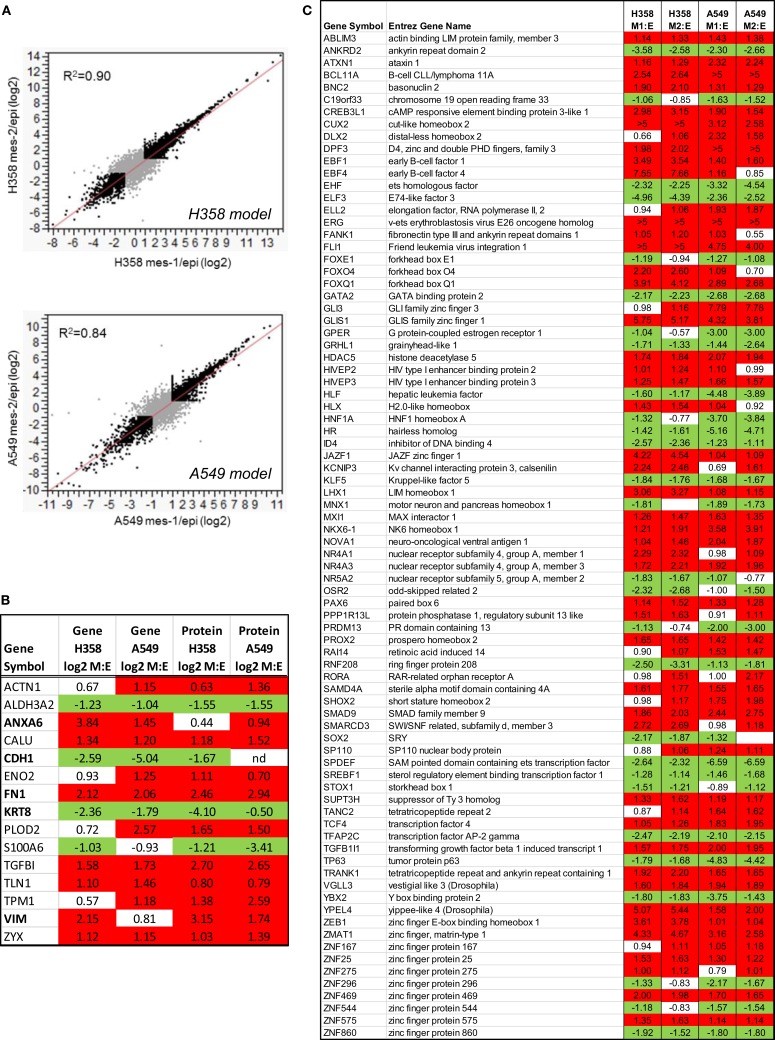
**(A)** Regression analysis of duplicate mesenchymal RNA-Seq samples expressed at a ratio to the mean epithelial control for both H358 and A549 models (greater than twofold changes are indicated in black). **(B)** Concordant RNA and protein changes for both isogenic H358 and A549 models. **(C)** Transcription associated RNA transcripts with differential abundance between epithelial and mesenchymal cell states in both H358 and A549 models.

In order the globally assess differences between isogenic epithelial and mesenchymal cell states, we measured RNA, protein, and phosphorylation changes as outlined in Figure 1E. RNA-Seq (44) was performed where non-zero ratios with RSEM reads ≥5 for any condition are listed in Table S1 in Supplementary Material (20,443 genes). Correlation between biological replicate RNA-Seq samples was *r*^2^ = 0.90 and *r*^2^ = 0.84 for H358 and A549 models, respectively (Figure 2A). Protein and phosphopeptide changes between epithelial and mesenchymal cell states were measured using SILAC labeling (31) in H358 and A549 TGFβ-treated or control. Analysis of RNA and protein abundance (Figure 2B) confirmed the gain of vimentin (VIM), fibronectin (FN1), and loss of keratin-8 (KRT8) and S100A6, expected RNA and protein changes occurring with mesenchymal trans-differentiation. We focused on changes in RNAs encoding transcription regulators, where 82 transcripts were coordinately regulated in comparing mesenchymal and epithelial cell states for both isogenic H358 and A549 models (Figure 2C).

### Functional annotation of transcription and epigenetic networks

Functional association with RNA abundance changes was assessed by GSEA. As expected multiple signatures associated with KRas transformation, stemness, and mesenchymal trans-differentiation were significantly enriched (Table S5 in Supplementary Material), with normalized enrichment score (NES) *p*-value <0.05 and the false discovery rate (FDR) *q*-value <0.05. Similarly pathway prediction analysis predicted activation of TGFβ signaling (45), and inhibition of DKK1 and SMAD7 signaling. These findings further served as validation benchmarks for the GSEA and pathway prediction approaches. Similarly benchmark RNA expression values for transcriptional regulators, which can induce EMT [Snail, Slug, Twist, and Zeb (9, 46–48)] were markedly increased in a model dependent manner (Table S6 in Supplementary Material).

Transcriptional signatures surrounding LEF1, NFκB, and BMI1 were highly enriched in datasets comparing H358 and A549 epithelial and mesenchymal-like cell states using GSEA and/or pathway prediction analysis (Table 1). We considered other predicted activated or inhibited nodes common to both KRas trans-differentiation models. Pathway prediction analysis was used to model signaling activation or inhibition based on transcription factor response. Table 2 summarizes pathway state based on H358 and A549 RNA-Seq datasets. Expected activation or Snail (H358), Twist (A549), and STAT pathways [H358 and A549 via increased IL11 and IL6; (23, 49)] was expected but highlight model heterogeneity, even within closely related NSCLC systems. Interestingly, RNA expression correlated the mesenchymal state with KDM5B, SMARCA4, and EGR1 pathway activation, and Myc/Max, SIN3A, and SPDEF pathway inhibition.

**Table 1:** **RNA changes correlating with gene-set enrichment (GSEA) signatures and/or pathway prediction analysis (IPA) for (A) Wnt/LEF1, (B) NFκB, and (C) BMI1 signaling networks in H358 and A549 KRas NSCLC models comparing isogenic epithelial and mesenchymal cell states**.

Node	Tool	Cell model	State	GSEA dataset NAME	Size	NES	NOM *p*-value	FDR *q*-value
**(A)**								
Wnt	GSEA	A549	Epi	LEF1_UP.V1_DN	186	1.62	*0.000*	*0.011*
		H358			178	1.66	*0.000*	*0.013*
	IPA			IPA transcription regulator	IPA predicted activation state	Reg. *z*-score	*p*-Value overlap	
		H358	Mes	TCF12	Activated	1.45	*0.040*	
		A549				0.87	*0.046*	
		H358		WISP2	Inhibited	-4.26	*0.021*	
		A549				-2.31	*0.000*	
**(B)**								
NFkB	GSEA	A549	Epi	HINATA_NFKB_TARGETS_DN	23	1.30	0.102	0.243
		H358			23	1.99	*0.006*	*0.005*
		A549	Mes	HINATA_NFKB_TARGETS_UP	91	-1.85	*0.000*	*0.013*
		H358			87	-1.53	*0.004*	0.121
		A549		SCHOEN_NFKB_SIGNALING	34	-1.57	*0.010*	0.149
		H358			33	-1.44	0.054	0.182
	IPA			IPA transcription regulator	IPA predicted activation state	Reg. *z*-score	*p*-Value overlap	
		H358		NFKB1	Activated	0.61	*0.002*	
**(C)**								
BMI	GSEA	A549	Epi	BMI1_DN.V1_DN	141	1.50	*0.000*	*0.023*
		H358			126	1.54	*0.000*	*0.017*
		A549	Mes	BMI1_DN.V1_UP	145	-1.49	*0.000*	*0.032*
		H358			142	-1.66	*0.000*	*0.007*
		A549	Epi	BMI1_DN_MEL18_DN.V1_DN	145	1.54	*0.000*	*0.016*
		H358			129	1.66	*0.000*	*0.015*
		A549	Mes	BMI1_DN_MEL18_DN.V1_UP	144	-1.82	*0.000*	*0.002*
		H358			141	-1.83	*0.000*	*0.001*
		A549	Mes	PRC1_BMI_UP.V1_DN	184	-1.30	*0.023*	0.119
		H358			158	-1.41	*0.004*	0.060
		A549		WIEDERSCHAIN_TARGETS_BMI1_PCGF2	57	-1.49	*0.011*	0.213
		H358			56	-1.56	*0.009*	0.104

*Individual GSEA signatures are defined in detail at http://www.broadinstitute.org/gsea/msigdb/index.jsp. Statistical significance is denoted by values in italics*.

**Table 2:** **Transcription pathway prediction (IPA) based on differential RNA expression between mesenchymal and epithelial cell states for both A549 and H358 models**.

Model	Upstream regulator	Predicted activation state	Activation *z*-score	*p*-Value of overlap
H358dT	E2F6	Inhibited	-2.55	0.002
A549	E2F6		-1.85	0.002
H358dT	EGR1	Activated	2.95	ns
A549	EGR1	Activated	3.14	ns
H358dT	ESRRA	Inhibited	-2.30	0.006
A549	ESRRA	Inhibited	-3.33	0.009
H358dT	ETS1		1.45	0.029
A549	ETS1		1.92	ns
H358dT	FOXM1	Inhibited	-2.70	0.014
A549	FOXM1		-1.19	0.023
H358dT	KDM5B	Activated	3.52	0.000
A549	KDM5B	Activated	2.27	0.000
H358dT	MAX	Inhibited	-3.20	0.012
A549	MAX	Inhibited	-2.18	0.002
H358dT	MYC	Inhibited	-3.76	0.000
A549	MYC	Inhibited	-2.16	0.000
H358dT	NRF1		-1.17	0.004
A549	NRF1	Inhibited	-3.03	0.024
H358dT	PPARGC1B		-0.96	0.003
A549	PPARGC1B		-0.61	0.046
H358dT	RUVBL1		-1.06	0.001
A549	RUVBL1		-1.92	0.000
H358dT	SIN3A	Inhibited	-2.33	ns
A549	SIN3A	Inhibited	-2.33	ns
H358dT	SMAD7	Inhibited	-2.02	ns
A549	SMAD7		-1.87	ns
H358dT	SMARCA4	Activated	2.67	ns
A549	SMARCA4	Activated	2.42	ns
H358dT	SNAI1	Activated	2.32	0.053
H358dT	SPDEF	Inhibited	-4.24	ns
A549	SPDEF	Inhibited	-2.92	0.027
A549	STAT3	Activated	2.10	ns
H358dT	STAT6		1.93	ns
H358dT	TP53	Activated	3.07	0.000
A549	TP53	Activated	3.07	0.000
H358dT	TRIM24	Activated	2.93	ns
A549	TRIM24		1.76	ns
H358dT	TSC22D1	Activated	2.24	ns
A549	TSC22D1	Activated	2.24	ns
H358dT	TWIST2		1.76	ns
A549	TWIST2	Activated	2.19	0.032
H358dT	USF1		1.88	ns
A549	USF1		1.89	ns
H358dT	WT1		0.84	0.003
A549	WT1		1.02	0.001
H358dT	XBP1	Activated	6.62	0.001
A549	XBP1		1.24	0.000
H358dT	YY1		1.77	0.003
A549	YY1		1.56	0.006

*Expected marker genes, Snail for H358 and Twist2 for A549, served as internal benchmarks. Activation of transcriptional regulators KDM5B, EGR1, SMARCA4, TP53, TSC22D1, inhibition of ESRRA, MYC/MAX, SIN3A, and SPDEF were correlated with both H358 and A549 mesenchymal states. TGFβ1, the EMT inducer, served as an internal control*.

### Altered TCF/LEF and NFκB networks with the mesenchymal cell state

**Figure 3:**
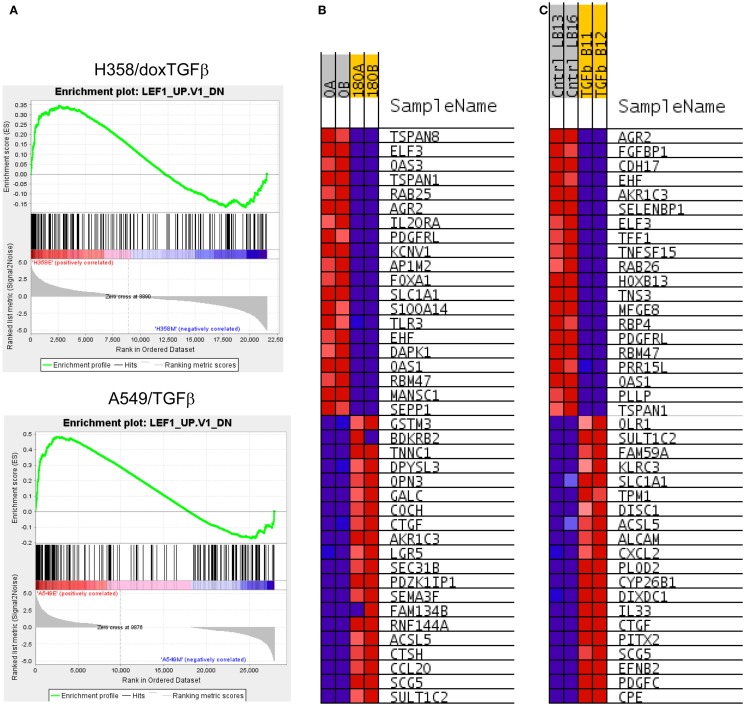
**(A)** Correlation of H358 and A549 EMT regulated genes with the LEF1 signature from LEF1 over-expressing epithelial DLD1 cells (from GSE3229). Normalized enrichment score (NES) was 2.63, nominal *p*-value, FDR *q*-value, and FWER *p*-value were <0.001. **(B)** The top 20 positively and negatively correlated genes were identified and heat mapped for H358 **(B)** control [0; A, B] and TGFβ [180; A, B] duplicate samples) and **(C)** for A549 (control [Cntrl; LB13, LB16] and TGFβ [TGFb; B11, B12] duplicate samples).

**Figure 4:**
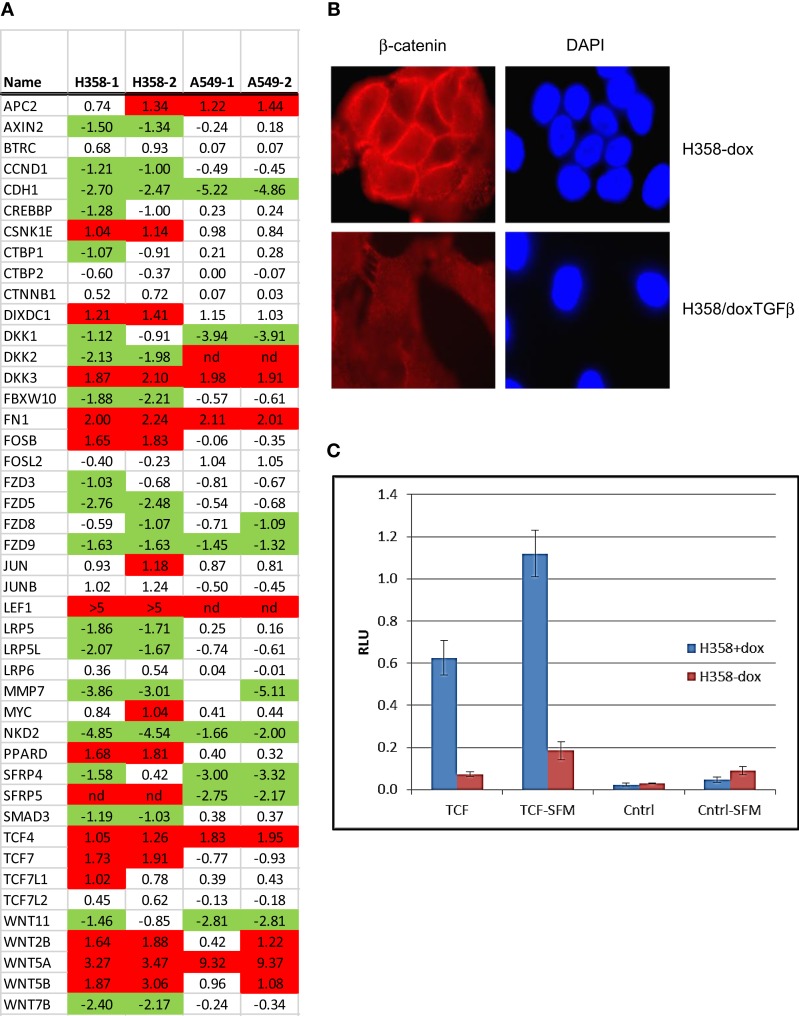
**Nuclear translocation of β-catenin and TCF/LEF activation in steady-state mesenchymal H358 cells expressing Wnt5A**. **(A)** Heat map of RNA abundance of Wnt signaling components and target genes in H358 and A549 isogenic models (mesenchymal/epithelial; log2) from duplicate samples. **(B)** Loss of membrane β-catenin localization and gain of punctate nuclear localization in mesenchymal H358/dox-TGFβ cells. Top panels: H358 cells in the absence of doxycycline. Bottom panels: H358/dox-TGFβ cells in a mesenchymal-like state. Cells were labeled with β-catenin antibody (red) and DAPI (blue) and imaged (60X). **(C)** Co-transfection of sTOP-TCF/LEF-luciferase (“TCF”) or control FOP-luciferase (“Cntrl”) was used to measure the activity of the TCF–LEF pathway. Renilla-luciferase was used to normalize transfection efficiency. H358/dox-TGFβ cells, in the presence or absence of doxycycline, were transfected and after 48 h luciferase measurements preformed under standard conditions. Both steady-state serum and 24 h serum starvation conditions (“-SFM”) were used with similar results. The *y*-axis units are relative light units (RLU). The means of two independent experiments are shown, each in triplicate, where the error bars reflect the standard error of the mean.

Canonical Wnt signaling leads to the nuclear translocation of β-catenin and the activation of TCF and LEF family transcription factors, which in turn promote pro-survival gene expression programs. The activation of the Wnt/β-catenin/TCF–LEF axis has been implicated in epithelial mesenchymal transition and metastatic behavior (50, 51). GSEA correlations predict LEF1 pathway activation in the mesenchymal state (Table 1). Figure 3A shows GSEA plots of genes down-regulated by overexpression of LEF1 in epithelial DLD1 cells (52), positively correlating with the epithelial cell state. Correlations between the H358 and A549 RNA abundance changes and LEF1 gene signatures were statistically significant (*p* < 0.0001 and FDR q-value <0.02; Table 1A). The top 20 positively and negatively correlated genes were identified and heat mapped for H358 (Figure 3B; control and TGFβ duplicate samples) and for A549 (Figure 3C; control and TGFβ duplicate samples). The data suggested TCF/LEF1 signaling were up-regulated in EMT-derived mesenchymal lung tumor cells. We asked whether EMT state in H358 or A549 cells might alter the abundance of RNA transcripts encoding components of the Wnt signaling pathway itself, where log2 ratios are shown for duplicate samples comparing 44 Wnt pathway components between mesenchymal and epithelial states (Figure 4A). Both positively acting and negatively acting Wnt pathway encoded RNAs were observed. The Wnt pathway positive regulators TCF4/TCF7L1/LEF1 and WNT5A/5B were found to be up-regulated in either H358 or A549 mesenchymal cell states, while the negative regulators DKK1 and NDK2 were down-regulated in mesenchymal cell states.

Nuclear β-catenin localization has been shown to correlate with neoplastic transformation and cancer patient outcome (53). In normal epithelial cells β-catenin forms a complex with α-catenin and E-cadherin on the inner side of the plasma membrane, important for cell–cell interaction. By immunofluorescence microscopy, we observed that β-catenin was localized at cell–cell-junctions on the inner side of the plasma membrane in the epithelial cell state, as expected (Figure 4B; top panels). In the mesenchymal cell state, β-catenin staining was lost from the cell membrane, with expected (54) diffuse cytoplasmic and speckled nuclear staining (Figure 4B; bottom panels). We asked whether signaling through the Wnt/β-catenin pathway might show a change in activity between epithelial and mesenchymal lung states. H358 cells were transfected with TCF promoter–reporter plasmid (sTOP), measuring TCF/LEF dependent transcription, or control (FOP) plasmid with mutated TCF/LEF binding sites. A control TK-renilla plasmid was used to control for transfection efficiency. Triplicate biological experiments were performed, each run in triplicate, which were normalized to the TK-renilla control and averaged (Figure 4C). We observed the signal from the TCF/LEF reporter was increased 8.5-fold in the mesenchymal state relative to the epithelial state in all experiments (*p* = 0.02). Similar findings were observed following serum removal for 24 prior to transfection and maintained for 48 h following transfection, where TCF/LEF reporter activity was increased in the mesenchymal state 6.1-fold (*p* = 0.004). The mutated control reporter showed minimal activity relative to TCF [*p* = 0.02 in serum and *p* = 0.002 in serum-free conditions (SFM)]. The use of LEF signatures and TCF/LEF reporter data serves in part to validate the informatics approach, further supported by correlation with expected gene signatures (Table S5 in Supplementary Material) for TGFβ, stem cell, and KRas pathways.

**Figure 5:**
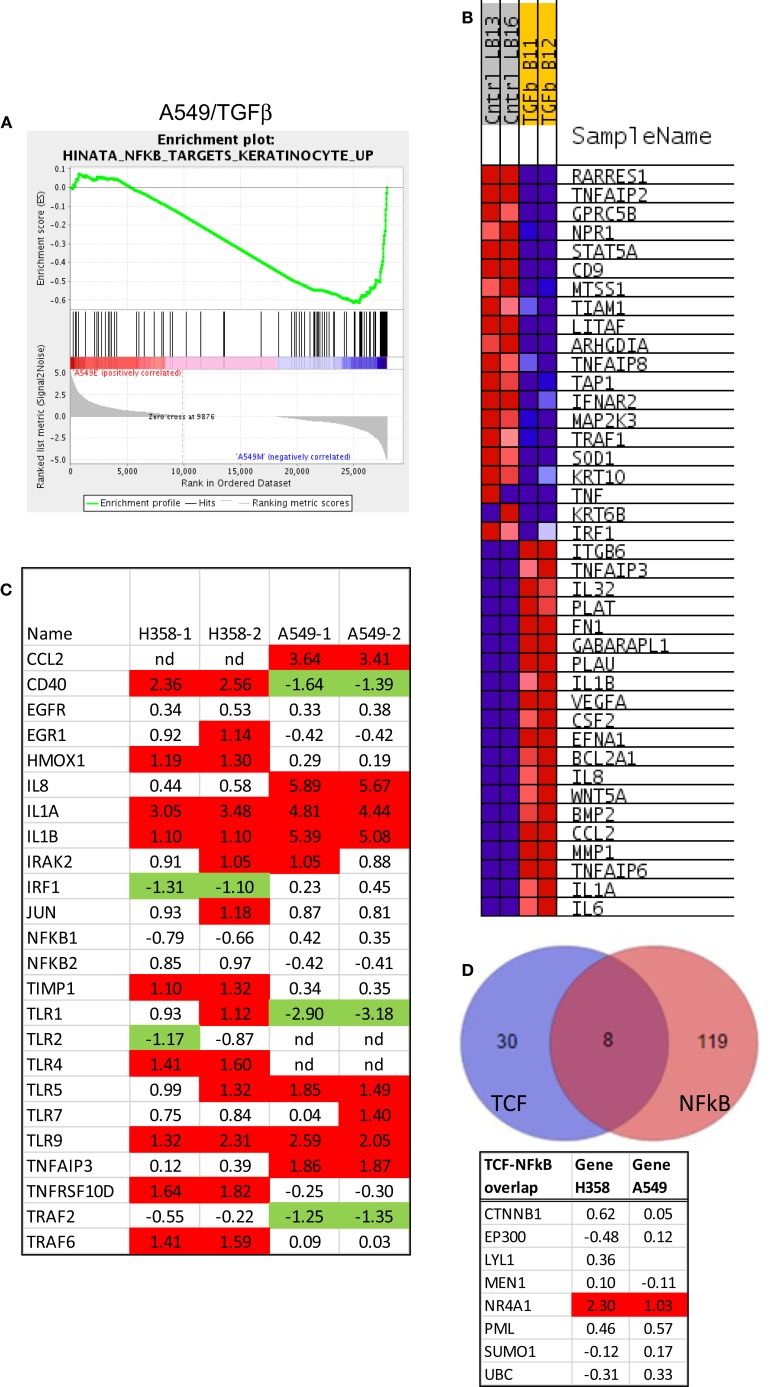
**Activation of the NFκB pathway in mesenchymal H358**. **(A)** Correlation of A549 EMT regulated genes with the NFκB signature from NFκB and RELA over-expression in keratinocytes (52). Normalized enrichment score (NES) was −1.85, nominal *p*-value <0.001, FDR *q*-value 0.01. **(B)** The top 20 positively and negatively correlated genes were identified and heat mapped for A549, as defined in Figure 4. **(C)** Heat map of RNA abundance of NFκB signaling components and target genes in H358 and A549 isogenic models (mesenchymal/epithelial; log2). **(D)** Protein–protein overlaps between TCF4 and NFκB protein interaction datasets from BioGrid, showing NR4A1 as a common physical node between the two pathways.

Gene-set enrichment and pathway prediction analysis of RNAs altered in abundance with mesenchymal trans-differentiation, indicated potential activation of the NFκB pathway, particularly in A549 (Table 1B; Figure 5A). The Hinata NFκB signature was derived from normal keratinocytes over-expressing NFKB1 and RELA (52). The Schoen NFκB signature reflects genes down-regulated in mesenchymal-like A375 melanoma cells treated with an NFκB inhibitor (55). The top 20 positively and negatively correlated genes form the Hinata signature correlation were identified and heat mapped for A549 (Figure 5B). RNAs encoding components of the pathway, notably IL1, TLR5, and TLR9, were markedly increased in the mesenchymal state (Figure 5C). It is beyond the scope of this study to fully define upstream signaling pathways, which promote the transcriptional and epigenetic changes observed in H358 and A549 models. However, network predictions from RNA sequencing datasets, phosphoproteomic analysis, and kinase activity immunoblots all suggest pronounced activation of ERK1/2 (56, 57) and casein kinase-2 (CK2) and the inhibition of glycogen synthase-3 (GSK3) protein kinase activities in both A549 and H358 mesenchymal states. Phosphorylation of GSK3beta on the kinase activating site Y216 is reduced in H358 mesenchymal cells (data not shown). The inhibition of GSK3 has been shown to stabilize Snail, which in turn increases Zeb1 expression, while CK2 kinase activity has been correlated with NFκB activation (58). Wnt5A is a transcriptional target of NFκB, which is increased in the mesenchymal state in both H358 and A549 models. Wnt5A driven TCF/LEF targets are known to include CD44 and Snail, further reinforcing the establishment of the mesenchymal state. Interaction nodes between TCF/LEF and NFκB pathways were mapped using protein–protein interaction data (BioGrid; Figure 5D). The orphan nuclear receptor NR4A1/TR3/Nur77 was identified as an up-regulated common component of the LEF1 and NFκB 1 protein–protein interaction networks. NR4A1 showed coordinate regulation with redox sensitive genes, e.g., TXNDC5, suggesting NR4A1 may regulate oxidative stress as has been observed in pancreas cancer models (59). Low NR4A1 levels have been correlated with cisplatin resistance (60). The elevated RNA abundance for NR4A1 in the mesenchymal state, suggest mesenchymal-like tumor cells may be more sensitive to cisplatin. Our previous studies have shown mesenchymal H358 and A549 cells are relatively resistant to EGFR (8, 61, 62) and IGF1R/IR (63) TKIs and to gemcitabine and paclitaxel, while retaining sensitivity to cisplatin *in vitro*.

### Altered transcription and epigenetic networks

**Figure 6:**
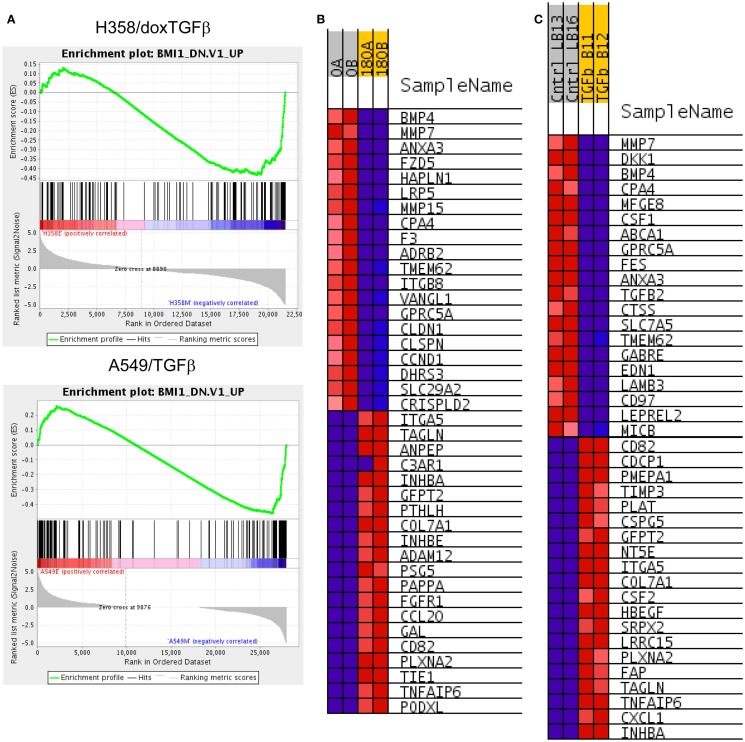
**(A)** Increased BMI target gene enrichment from RNA-Seq datasets comparing differential RNA expression between H358 and A549 cell states. Statistical significance (*p* < 0.0001 and FDR *q*-value <0.04 was observed. **(B)** The top 20 positively and negatively correlated genes were identified and heat mapped for H358 and **(C)** for A549, with labels as defined in Figure 3.

BMI1 is a component of the polycomb repressive complex (PRC1), formed by complexes with heterochromatin adaptor family proteins (e.g., CBX8). PRC1 functions as an inhibitor of transcription during embryogenesis and in neoplastic transformation, as a tumor suppressor. BMI1 is required for Twist mediated EMT (64). Multiple GSEA signatures associated with changes in BMI1 target gene expression were observed (Table 2C), with the majority showing stringent statistical significance (*p* < 0.05 and FDR *q*-value <0.05). The BMI_DN and Wiederschain signatures reflect genes up-regulated the stem-like medulloblastoma line DAOY by knockdown of BMI1 with or without co-knockdown of the polycomb ring finger gene PCGF2/Mel-18 (65). In contrast, the PRC1_BMI signature (66) is derived by knockdown of BMI1 in fibroblasts, cells of mesodermal origin, showed a reversed correlation. The GSEA plots correlating BMI1 knockdown with the H358 and A549 mesenchymal cell state are shown (Figure 6A). The top 20 positively and negatively correlated genes were identified and heat mapped for H358 (Figure 6B) and for A549 (Figure 6C), suggesting altered regulation of the BMI1 pathway with EMT in the two mutant-KRas NSCLC models. Interestingly, the GSEA indicated better correlation with inhibition of BMI1 target genes with the mesenchymal cell phenotype. However, direct analysis of up-regulated and down-regulated BMI1 target genes showed enrichment in both H358 and A549 epithelial and mesenchymal cell states (Table S7 in Supplementary Material), which also was observed by GSEA (anti-correlation) at lower statistical stringency. These findings suggest additional factor(s) likely contribute to BMI1 target gene recognition and/or the directionality of regulation between epithelial and mesenchymal cell states. Non-PRC1 components may modify the directionality BMI1 output, for example, CtBP, E2F6, NFκB, and Cited2 (67).

**Figure 7:**
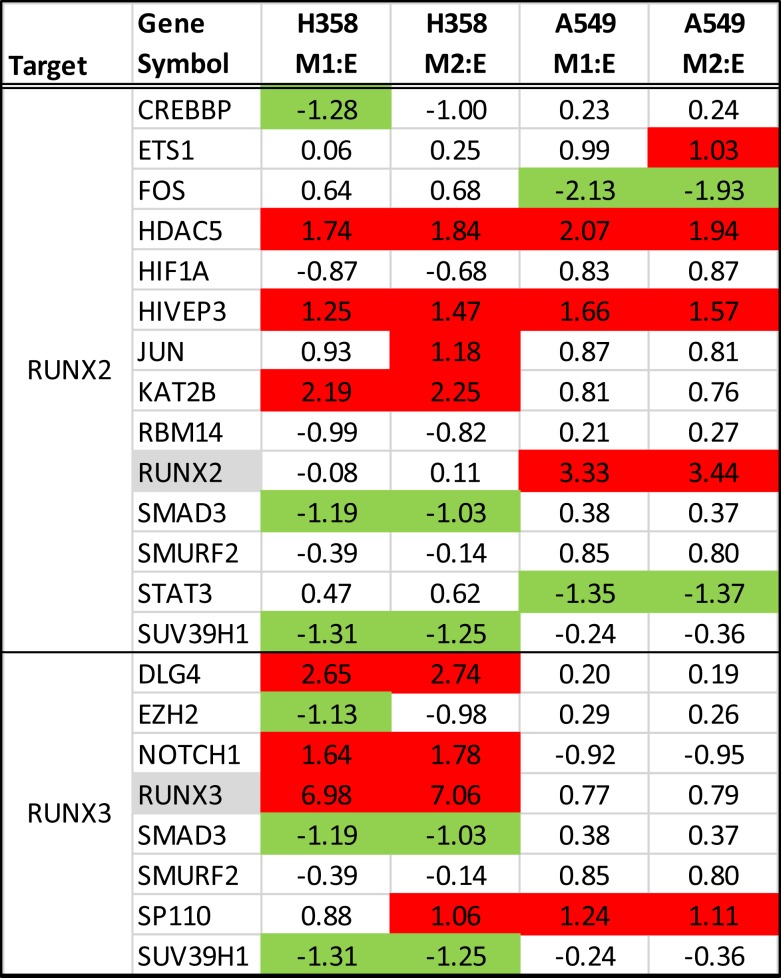
**RNA expression ratios comparing H358 and A549 mesenchymal/epithelial cell states in duplicate**. Complexes associated with RUNX2 (top) and RUNX3 (bottom) are shown.

Runx family transcription factors contain a conserved Runt DNA binding domain and are developmentally regulated. RUNX2 and RUNX3 are overexpressed in the A549 and H358 models, respectively. RUNX3, previously thought to function as a tumor suppressor, has recently been associated with cancer progression (68) and cooperative induction with NFκB of the inflammatory cytokine IL23 (69). In NSCLC overexpression of RUNX2 has been observed in comparisons of tumor and normal tissues and was implicated with poor outcome (70). The overexpression of exogenous RUNX2 also has been shown to promote EMT (71, 72), increase migratory and invasive behavior (73), and increase expression of Twist and Slug, both of which are markedly increased with EMT in the A549 background. We examined interaction networks around RUNX2 and RUNX3, where proteins forming direct contacts and potentially establishing signaling complexes, are shown in Figure 7. In H358 RUNX3 RNA expression was correlated with increased expression for potential protein–protein interactors SP110, NOTCH1, DLG4, JUN, KAT2B, HIVEP3, and HDAC5 and decreased expression and potential interaction with EZH2, SUV39H1, RBM14, CREBBP, and SMAD3. This also was associated with an increased SP110 phosphorylation of S256. In A549, Runx2 expression correlated with increased KAT2B, HIVEP3, ETS1, SP110, and HDAC5 expression and decreased expression of STAT3 and FOS.

Multiple transcription factors associated with the maintenance of the epithelial state were markedly decreased with EMT in both models. Grainyhead-like 1 (GRHL1) RNA expression is decreased in both mesenchymal models and GRHL2 is reduced in the H358 model. GRHL2 and GRHL1 share ~70% homology and GRHL2 has been associated with the maintenance of an epithelial state (74, 75), as a target repressed by Zeb1. Similarly transcription factor AP-2 gamma (TFAP2C), a transcription factor associated with estrogen receptor signaling in breast cancer (76), showed reduced expression in the mesenchymal state. Emerging data indicate TFAP2C is important for the normal luminal epithelial differentiation (77) where knockdown promotes mesenchymal transition (78). Conversely, overexpression of the TFAP2C cDNA reduced CD44 expression, and high TFAP2C expression was correlated with response to neoadjuvant chemotherapy (79). EHF, an ETS family transcription factor, also has been associated with an epithelial differentiation program and EHF RNA expression was decreased (74). Interestingly, the epithelial-specific splicing factors ESRP1 and ESRP2 (80) are markedly attenuated with EMT in the H358 model, but their expression is absent in both epithelial and mesenchymal A549 states, again indicating considerable heterogeneity in EMT programs within closely related NSCLC models. Finally, Odd-skipped related 2 (OSR2) is a Smad3/4 down-regulated palate and limb developmental gene (81), reduced in the both mesenchymal models.

Epithelial mesenchymal transition state was correlated with statistically significant changes in site-specific phosphorylation (Table 3). Several members of the BAF SWI/SNF complex chromatin remodeling proteins were altered in their pattern of site-specific phosphorylation dependent on EMT state. Notably SMARCC1 showed increased phosphorylation at positions S328 and S330, and ARID1A was increased in phosphorylation at position S696. CBX3/HP1γ is a chromatin remodeling factor implicated in euchromatin silencing in embryonic stem cells (82) and in transcription elongation by RNA polymerase II on heterochromatic genes (83). CBX3/HP1γ has shown overexpression in NSCLC (84) as compared with normal adjacent tissue, and expression was correlated with poor survival rate [*p* = 0.02; (85)]. Phosphorylation measurements also suggested EMT state may be regulated in part by increased phosphorylation of CBX3/HP1 at position S95 (*p* < 0.001) and likely S93 (*p* = 0.10; not shown) in the central region between chromo and chromoshadow domains. CBX3/HP1 functions as a transcriptional silencer through histone H3 K9 interaction, where phosphorylation at S93, likely by PKA, results in a reduction of CBX3/HP1 mediated silencing and transcription elongation (86). Change in phosphorylation of CBX8, a component of the PRC1 complex, at position S191 within the atrophin-1 domain also was observed (Table 3). Phosphorylation of HDAC2 was increased at positions S422 and S424 in the mesenchymal state. S422 and S424 are known CK2a sites, associated with inhibition of deacetylase activity (87). S394 is also a CK2a phosphosite increased in H358, decreased in A549. The reported role S394 in activation (88) and inhibition (87) of HDAC2 may be context dependent through as yet undefined factors.

**Table 3:** **Statistical analysis of phosphopeptide changes with EMT state, from proteins specifically associated with transcriptional function (where *N* ≥ 4)**.

Cell model	Gene symbol	Site	*N*	Peptide log2 M:E	*p*-Value
H358	ARID1A	S696	8	0.67	0.0001
A549	BAZ1B	S1468	6	−0.20	0.0001
H358	BRD3	S263	4	1.06	0.0016
H358	CBX3	S95	17	0.70	0.0001
H358	CBX8	S191	4	−0.59	0.0036
H358	CTR9	S970	5	0.84	0.0001
A549	CTR9	T925	6	0.56	0.0009
H358	CTR9	T925	7	−0.05	0.0862
H358	DAXX	S495	14	0.84	0.0001
H358	DPF2	S142	7	0.35	0.0009
H358	EAF1	S158, S165	5	1.12	0.0001
H358	FLII	S856	4	0.50	0.0001
H358	FOXK1	S416, S420	17	0.45	0.0001
H358	FOXK1	S441, S445	6	0.02	0.0001
A549	HDAC1	S393	15	−0.35	0.0001
H358	HDAC1	S393	10	0.02	0.0008
A549	HDAC2	S394	22	−0.12	0.0001
H358	HDAC2	S394	7	0.31	0.0013
H358	HDAC2	S422, S424	18	0.36	0.0001
H358	HIRIP3	S125	12	0.01	0.0010
H358	HIRIP3	S223, S227	14	−0.23	0.0038
H358	HIRIP3	T84, S87	4	0.12	0.0022
H358	HIST1H1B	S18	54	−1.98	0.0001
H358	HIST1H1E	T18	46	−1.75	0.0001
H358	IRF2BP1	S384	8	0.50	0.0001
A549	IRF2BP1	S436	5	−0.05	0.0001
A549	IRF2BP2	S175	6	−0.97	0.0060
H358	IRF2BP2	S175	6	−0.83	0.0001
H358	LMO7	S1510	6	−3.39	0.0001
H358	MED1	T1051	7	−0.10	0.0151
H358	MEF2D	S231	5	1.16	0.0001
A549	MYBBP1A	S1267	6	−0.29	0.0001
H358	NCOR2	S956	23	1.01	0.0001
H358	PELP1	S481	11	0.55	0.0001
H358	PHC3	T609, S616	5	1.09	0.0017
A549	PNN	S100	14	−0.33	0.0001
H358	PNN	S100	45	0.63	0.0001
H358	PURB	S101	7	0.56	0.0001
H358	PURB	S304	15	0.53	0.0001
H358	RBM15	S670, S674	8	0.07	0.0001
A549	RBM15	T568	8	−0.02	0.0001
H358	RBM15	T568	4	0.18	0.0013
H358	SAFB	S601, S604	10	0.96	0.0001
H358	SAFB	S604	7	0.32	0.0002
H358	SMARCA5	S66	26	−0.48	0.0897
H358	SMARCC1	S328, S330	8	0.91	0.0001
H358	SNW1	S224, S232	12	0.22	0.0001
H358	SP110	S256	16	0.16	0.0001
H358	SSRP1	S437	57	0.04	0.0001
H358	SUDS3	S234, S237	13	0.07	0.0001
H358	TLE3	S263, S267	6	0.64	0.0002
H358	TRIM28	S19	34	0.52	0.0001
H358	TRIM28	S473	6	0.30	0.0001
H358	TRIM28	S33	6	0.19	0.0001
H358	YBX1	S165	4	0.59	0.0001
H358	YBX1	S167	16	0.58	0.0001
H358	YBX1	S174	10	0.67	0.0001
H358	YBX1	S176	4	0.63	0.0001
H358	YBX1	S2	5	0.24	0.0069
H358	ZC3H8	S77	4	1.19	0.0001

*Data are expressed as the log2 ratio of mesenchymal/epithelial values (log2 M:E). Log2 ratio values are colored where red denotes increased in the mesenchymal state and green denotes decreased in the mesenchymal state*.

The PAF1 complex protein CTR9/SH2BP1, which plays a role in the maintenance of ESC pluripotency showed phosphosite regulation at T925 (A549) and S970 (H358) (Table 3). Additional transcriptional and epigenetic regulators were found to be modified by phosphorylation, including NCOR2/SMRT, MEF2D, FOXK1, BRD3, and the PRC1 component PHC3. Decreased phosphorylation of the bromodomain protein kinase BAZ1B/WSTF at position S1468, increased phosphorylation of EAF1 at sites S158 and S165 were observed. The phosphorylation of TRIM28/KAP1 S473, likely by CHK2, inhibits co-repression of CDKN1A/p21 (89), and may contribute the observed attenuation of cell cycling. ZC3H8 functions as a repressor of GATA3 and shows increased phosphorylation at position S77 in mesenchymal H358 cells, where GATA3 has been associated with the TGFβ growth suppression response (90).

### Early transcriptional events in the trans-differentiation of mutant-KRas NSCLC models

We asked whether key transcription factors, highlighted by GSEA correlations, might show coordinate early expression during mesenchymal transition. RNA samples were isolated from the H358 model following TGFβ induction at 0, 1, 2, 4, 8, 16, 24, 72 h, 7 days (~168 h), 21 days (~500 h), and the long term/steady-state condition (>4500 h), and duplicate samples sequenced. TGFβ1 served as an internal control, where expression was observed within 1 h after addition of doxycycline.

The exchange of cell cycle inhibition with migratory and invasive gene activation programs is a hallmark of metastable mesenchymal trans-differentiation. Reduced RNA expression of cell cycle activators and increased expression of inhibitors was observed and served as a benchmark (Table 4). Early in the EMT epigenetic reprograming, inhibition of E2F family, MCM family, CCNE1 and CDCA7L gene products, and an increase in BTG2 expression was observed. This was accompanied by reduced phosphorylation of Histone H1B and H1E at positions S18 and T18, respectively (Table 3). Both sites are cell cycle sensitive, where T18 is a known CDK1 and CDK2 site.

**Table 4:** **Time course of cell cycle regulator RNA expression, comparing H358 mesenchymal–epithelial RNA abundance (log2 ratios)**.

Gene symbol	Name	Time (h)											
		0	1	2	4	6	8	18	24	72	168	500	4500
BTG2	BTG family, member 2	0.00	−0.14	0.09	0.29	0.57	0.33	1.46	1.14	1.45	1.17	0.77	0.82
CCNE1	Cyclin E1	0.00	−0.12	−0.10	−0.22	0.11	0.10	−1.67	−0.79	−1.98	−3.35	−0.65	0.35
CDCA7L	Cell division cycle associated 7-like	0.00	0.07	−0.01	−0.42	−0.23	−0.30	−1.48	−0.96	−3.14	−4.34	−1.91	−0.61
E2F1	E2F transcription factor 1	0.00	0.14	0.08	−0.34	−0.13	−0.24	−1.06	−0.64	−5.73	−6.26	−2.71	−0.77
E2F2	E2F transcription factor 2	0.00	0.06	0.09	−0.28	−0.22	−0.30	−1.69	−1.04	−4.77	−5.36	−3.68	−2.20
E2F8	E2F transcription factor 8	0.00	−0.03	0.11	−0.14	−0.11	0.01	−1.25	−0.91	−4.07	−6.14	−3.23	−0.48
MCM2	Minichromosome maintenance complex component 2	0.00	0.12	0.06	−0.38	−0.04	−0.10	−1.15	−0.69	−3.89	−4.12	−2.10	−0.74
MCM4	Minichromosome maintenance complex component 4	0.00	0.05	0.04	−0.26	−0.07	−0.13	−1.09	−0.68	−4.34	−4.56	−2.37	−0.94
MCM5	Minichromosome maintenance complex component 5	0.00	0.08	0.03	−0.38	−0.11	−0.20	−1.12	−0.51	−3.87	−3.36	−2.03	−0.95
MCM6	Minichromosome maintenance complex component 6	0.00	0.04	0.05	−0.23	−0.10	−0.15	−1.06	−0.49	−2.87	−3.89	−2.75	−0.84
RBL1	Retinoblastoma-like 1	0.00	−0.01	0.17	−0.30	−0.16	−0.15	−1.27	−0.84	−3.62	−2.92	−2.00	−0.71
TGFB1	Transforming growth factor, beta 1	0.00	0.61	0.94	1.10	1.92	2.00	2.80	2.90	3.69	4.82	3.25	4.63

*Genes were selected based on expression in 6–8 or 18–24 h time bins. TGFβ1, the doxycycline-induced transgene, served as an internal control. Log2 ratio values are colored where red denotes increased in the mesenchymal state and green denotes decreased in the mesenchymal state*.

We then examined changes in RNA abundance for encoded transcriptional and epigenetic regulators. We identified 81 transcriptional and epigenetic regulators expressed at early time points in primarily two time bins (6–8 and 18–24 h; Table 5). ELF3, ATOH8, EAF2, MYC, IRX5, CITED2, and ID2 showed relatively rapid repression ~8 h after induction, which was continued at later time points. ELF3 encodes an E74-like domain transcription factor, which is epithelial-specific. FOXS1, STAT5A, and HIC1 were increased at the early, continuing on to later time points. By 18–24 h HR, FOXR2, SOX2, RORA, and VSX1 showed a decrease in RNA abundance, while FOXS1, DMBX1, CDMBX1, CITED4, RORC, VGLL3, RUNX3, and BHLHE40 were markedly increased. The homeodomain transcription factor DMBX1 has been shown to be required for reprograming of induced pluripotent stem cells, and for midbrain development (91). Several transcription regulators showed biphasic expression, where an early decrease in RNA abundance was followed by an increase at the >6 months steady-state time point for the helix-loop-helix dominant negative E-box binding inhibitor ID2 and RAR-related orphan receptor RORA, while the reverse was true for PBX/knotted 1 homeobox 2 (PKNOX2), which was increased at early time points and markedly attenuated by 21 days (Table 5).

**Table 5:** **Time course of transcription factor RNA expression, comparing H358 mesenchymal–epithelial RNA abundance (log2 ratios)**.

Gene symbol	Name	Time (h)											
		0	1	2	4	6	8	18	24	72	~168	~500	>4500
ARID5A	AT rich interactive domain 5A (MRF1-like)	0.00	−0.01	−0.22	−0.41	0.05	−0.04	0.42	0.98	1.04	1.20	1.05	1.33
ATF5	Activating transcription factor 5	0.00	0.26	0.19	−0.19	0.20	0.06	−1.06	−0.55	−1.32	−1.94	−0.52	−1.54
ATOH8	Atonal homolog 8	0.00	−0.12	−0.23	−1.06	−0.66	−0.78	−2.00	−2.35	−3.91	−3.64	−3.91	
BHLHE22	Basic helix-loop-helix family, member e22	0.00	−0.12	−0.16	−0.49	0.10	−0.14	−1.58	−0.64	−2.06	−2.08	−1.12	−2.22
BHLHE40	Basic helix-loop-helix family, member e40	0.00	−0.26	−0.23	−0.49	−0.17	−0.02	1.22	1.30	2.04	1.89	0.81	1.42
CHAF1A	Chromatin assembly factor 1, subunit A	0.00	0.04	−0.01	−0.31	−0.09	−0.18	−1.34	−0.69	−3.72	−3.74	−1.96	−0.82
CHAF1B	Chromatin assembly factor 1, subunit B	0.00	0.14	0.08	−0.24	−0.07	−0.07	−1.17	−0.64	−4.07	−4.34	−2.64	−1.26
CITED2	Cbp/p300-interacting transactivator 2	0.00	0.24	−0.13	−0.61	−0.23	−0.50	−1.19	−0.76	−0.56	−0.57	−0.53	−1.23
CITED4	Cbp/p300-interacting transactivator 4	0.00	0.20	0.12	−0.41	0.06	0.22	1.73	1.45	2.53	3.83	2.27	−0.18
CREB3L1	cAMP responsive element binding protein 3-like 1	0.00	0.01	0.12	−0.34	0.07	0.11	1.00	1.46	2.23	1.04	2.04	3.07
DMBX1	Diencephalon/mesencephalon homeobox 1	0.00	−0.03	0.02	−0.25	−0.21	−0.11	1.84	1.92	3.07	3.31	2.24	1.66
DPF1	D4, zinc and double PHD fingers family 1	0.00	−0.02	−0.24	−0.62	0.11	0.14	−1.04	−0.27	−1.58	−2.91	−0.50	−0.19
EAF2	ELL associated factor 2	0.00	−0.11	−0.41	−0.48	−0.53	−0.58	−1.43	−0.51	−0.65	−1.86	−2.36	−0.61
ELF3	E74-like factor 3	0.00	0.45	0.00	−0.95	−0.55	−0.93	−1.36	−1.46	−2.34	−1.32	−1.95	−4.68
ELK3	ELK3, ETS-domain protein (SRF accessory protein 2)	0.00	−0.01	−0.04	−0.24	−0.01	−0.03	0.59	0.92	1.62	1.19	1.00	1.19
FOXR2	Forkhead box R2	0.00	−0.01	−0.04	−0.26	0.06	−0.03	−2.02	−1.13	−4.27	−3.89	−1.12	−1.18
FOXS1	Forkhead box S1	0.00				0.98	0.93	2.19	3.29	5.44	3.47	5.80	6.11
GPS2	G protein pathway suppressor 2	0.00	0.03	−0.08	−0.42	−0.03	−0.08	−1.18	−0.71	−1.03	−0.78	−0.07	−0.18
GRHL3	Grainyhead-like 3	0.00	0.24	0.23	−0.45	−0.06	0.17	−1.13	−0.54	−1.19	−1.07	−1.86	0.41
HIC1	Hypermethylated in cancer 1	0.00	0.26	0.27	−0.31	0.23	0.33	1.45	2.20	2.44	2.04	1.98	2.84
HR	Hairless homolog	0.00	0.05	−0.06	−0.50	0.16	0.26	−2.39	−1.20	−2.06	−3.33	−2.27	−1.51
ID2	Inhibitor of DNA binding 2	0.00	0.27	0.16	−0.84	−0.48	−0.46	−1.69	−1.01	−1.11	−1.67	−2.65	2.24
ID3	Inhibitor of DNA binding 3	0.00	−0.06	−0.04	−0.64	−0.27	−0.37	−1.63	−1.07	−2.19	−3.75	−3.35	−0.86
ID4	Inhibitor of DNA binding 4	0.00	0.34	0.17	−0.53	−0.16	−0.29	−1.65	−1.58	−1.06	−0.86	−1.73	−2.46
ING3	Inhibitor of growth family, member 3	0.00	0.12	0.16	−0.29	−0.01	−0.08	−1.01	−0.59	−1.26	−1.21	−0.63	−0.68
IRF6	Interferon regulatory factor 6	0.00	0.01	−0.15	−0.36	−0.17	−0.17	1.20	1.25	2.48	1.55	0.42	0.61
IRX5	Iroquois homeobox 5	0.00	−0.09	0.02	−0.47	−0.23	−0.52	−1.25	−0.66	−1.39	−1.21	−1.84	−3.58
ISL1	ISL LIM homeobox 1	0.00	0.06	−0.15	0.06	0.17	0.21	−1.58	−0.41	−1.08	−1.63	−1.00	0.09
JDP2	Jun dimerization protein 2	0.00	−0.09	−0.14	−0.18	0.28	0.13	1.01	1.70	3.85	3.33	2.35	0.98
JUNB	Jun B proto-oncogene	0.00	−0.09	0.03	−0.87	0.06	0.19	1.13	1.58	1.95	2.31	1.46	1.14
KDM5B	Lysine (K)-specific demethylase 5B	0.00	−0.06	−0.20	−0.19	−0.12	−0.18	0.57	0.69	1.23	1.24	1.14	1.22
KDM6A	Lysine (K)-specific demethylase 6A	0.00	0.02	−0.11	−0.13	−0.15	−0.20	−0.21	0.04	−1.15	−1.70	−1.17	−1.92
KDM6B	Lysine (K)-specific demethylase 6B	0.00	−0.18	−0.29	−0.15	0.07	−0.05	0.83	0.91	1.80	2.22	1.27	1.43
LMCD1	LIM and cysteine-rich domains 1	0.00	0.15	0.14	−0.35	−0.04	0.04	0.63	1.39	1.56	1.56	2.47	1.98
MECOM	Ecotropic viral integration site 1	0.00	0.08	0.09	−0.35	−0.01	0.02	−1.27	−0.62	−1.08	−1.51	−0.80	−1.22
MYB	v-myb Myeloblastosis viral oncogene homolog	0.00	−0.15	0.09	−0.07	−0.07	−0.22	−1.29	−0.67	−3.44	−3.70	−2.07	−1.04
MYBL2	v-myb Myeloblastosis viral oncogene homolog-like 2	0.00	0.09	0.07	−0.29	−0.06	−0.09	−1.07	−0.63	−6.49	−8.62	−4.42	−0.89
MYC	v-myc Myelocytomatosis viral oncogene homolog	0.00	0.39	−0.22	−0.77	−0.22	−0.54	−1.06	−0.47	−1.78	−1.65	−0.71	0.95
NCOA5	Nuclear receptor coactivator 5	0.00	0.01	−0.03	−0.46	−0.13	−0.13	−1.32	−0.44	−1.24	−0.88	−0.11	−1.36
NFATC2	Nuclear factor of activated T-cells 1	0.00	0.54	−0.14	−0.27	−0.02	0.26	0.50	1.18	1.57	1.54	1.85	3.17
NFATC4	Nuclear factor of activated T-cells 4	0.00	−0.08	−0.28	−0.27	−0.06	−0.32	1.19	0.89	2.01	2.36	1.08	1.54
NME1	Non-metastatic cells 1, protein NM23A	0.00	0.09	−0.04	−0.41	−0.07	−0.08	−1.08	−0.60	−2.51	−2.78	−1.13	−0.75
NR4A1	Nuclear receptor subfamily 4, group A, member 1	0.00	−0.29	−0.23	−0.01	0.24	0.16	0.88	1.35	2.27	1.94	0.32	2.33
NRL	Neural retina leucine zipper	0.00	0.45	0.29	−0.19	0.22	0.10	−1.36	−0.86	−1.33	−0.71	−0.78	−0.81
PKNOX2	PBX/knotted 1 homeobox 2	0.00	−0.24	−0.32	−0.81	−0.25	−0.29	1.29	0.84	2.05	1.68	−1.99	−3.37
PPARD	Peroxisome proliferator-activated receptor delta	0.00	0.04	−0.15	−0.43	−0.06	−0.18	0.92	1.12	2.90	2.77	1.70	1.76
PPARGC1A	PPAR gamma, coactivator 1 alpha	0.00	−0.16	−0.02	−0.53	−0.07	−0.16	−1.46	−1.27	−0.56	0.09	−0.23	0.89
PRDM13	PR domain containing 13	0.00	0.12	0.03	−0.81	−0.20	−0.11	−1.61	−0.83	−2.42	−2.54	−1.42	−0.93
PSMC3	Proteasome 26S subunit, ATPase, 3	0.00	0.11	0.03	−0.33	−0.02	−0.01	−0.83	−0.45	−1.08	−1.26	−0.75	−0.44
PSMC3IP	PSMC3 interacting protein	0.00	0.08	0.00	−0.40	0.05	−0.08	−1.12	−0.66	−4.29	−3.67	−1.48	−0.27
RORA	RAR-related orphan receptor A	0.00	−0.32	−0.23	−0.24	−0.02	−0.15	−1.70	−1.38	−1.65		−2.14	1.25
RORC	RAR-related orphan receptor C	0.00	0.58	0.47	−0.33	0.32	0.26	1.55	1.93	3.88	2.64	2.30	−0.67
RUNX3	Runt-related transcription factor 3	0.00	0.07	−0.02	−0.35	0.31	0.27	1.27	1.63	2.37	1.80	1.04	7.04
SAP30	Sin3A-associated protein, 30 kDa	0.00	0.14	0.04	−0.17	0.10	0.02	−1.02	−0.68	−1.76	−1.81	−0.81	−0.47
SIRT4	Sirtuin	0.00	0.01	−0.48	−0.20	0.17	0.12	1.18	1.00	3.10	2.87	2.01	2.45
SIVA1	SIVA1, apoptosis-inducing factor	0.00	0.02	−0.10	−0.54	−0.11	−0.11	−1.10	−0.56	−1.43	−2.25	−0.95	−1.51
SKIL	SKI-like oncogene	0.00	−0.09	0.13	−0.48	−0.21	−0.02	1.16	1.04	2.10	2.52	1.23	1.47
SMAD7	SMAD family member 7	0.00	0.23	−0.05	−0.76	−0.14	0.04	0.80	1.03	2.04	2.13	1.38	0.93
SOX2	SRY -box 2	0.00	−0.02	−0.29	−0.36	−0.15	−0.44	−1.75	−1.37	−3.08	−2.71	−3.41	−2.02
SP6	Sp6 transcription factor	0.00	0.58	0.15	−0.04	0.18	−0.29	1.14	1.06	2.14	1.64	1.39	2.83
STAT5A	Signal transducer and activator of transcription 5A	0.00	−0.15	0.27	0.55	0.24	0.44	2.19	1.02	3.78	3.75	1.30	3.64
TADA2A	Transcriptional adaptor 2 -like	0.00	−0.16	0.03	−0.41	−0.15	−0.10	−1.13	−0.56	−1.33	−1.53	−0.78	−0.52
TCF19	Transcription factor 19	0.00	0.00	−0.01	−0.30	−0.02	−0.02	−1.10	−0.44	−3.62	−4.45	−2.23	−0.99
TFAP4	Transcription factor AP-4	0.00	−0.08	−0.17	−0.35	−0.06	−0.11	−1.13	−0.67	−1.29	−1.74	−1.01	−0.57
TGFB1	Transforming growth factor, beta 1	0.00	0.61	0.94	1.10	1.92	2.00	2.80	2.90	3.69	4.82	3.25	4.63
TP53INP1	Tumor protein p53 inducible nuclear protein 1	0.00	0.08	0.02	−0.14	−0.50	−0.34	1.14	1.14	3.98	4.39	2.37	1.94
TP73	Tumor protein p73	0.00	−0.01	−0.02	−0.31	−0.14	−0.27	0.95	1.33	2.57	2.15	1.98	1.84
UHRF1	Ubiquitin-like with PHD and ring finger domains 1	0.00	0.06	0.06	−0.32	−0.08	−0.13	−1.20	−0.55	−3.44	−3.82	−1.60	−1.25
VGLL3	Vestigial like 3	0.00	0.05	0.19	−0.31	−0.11	0.03	1.32	1.46	2.53	2.47	2.15	1.74
VSX1	Visual system homeobox 1	0.00	0.15	−0.18	0.02	0.16	−0.20	−1.64	−1.98	−1.58	−1.16	0.51	−1.26
YY2	YY2 transcription factor	0.00	0.04	0.04	−0.24	−0.03	−0.02	−1.12	−0.73	−1.86	−0.72	−0.14	−1.70
ZCCHC12	Zinc finger, CCHC domain containing 12	0.00	−0.12	−0.20	−0.32	−0.27	−0.06	−1.25	−1.00	−2.01	−1.12	−1.03	−2.38
ZFP112	Zinc finger protein 112 homolog	0.00	−0.22	−0.31	−0.21	0.03	−0.17	−1.30	−0.70	−1.70	−1.98	−0.63	0.15
ZFP90	Zinc finger protein 90 homolog	0.00	−0.09	−0.08	−0.24	−0.04	−0.02	−1.40	−0.94	−1.43	−2.11	−1.02	−1.66
ZNF180	Zinc finger protein 180	0.00	−0.05	0.03	−0.26	−0.04	−0.08	−1.01	−0.69	−1.92	−1.60	−0.89	−0.81
ZNF26	Zinc finger protein 26	0.00	0.03	0.07	−0.20	0.11	−0.08	−1.10	−0.53	−1.26	−1.02	−0.22	−0.35
ZNF296	Zinc finger protein 296	0.00	0.16	0.15	−0.59	0.28	0.08	−1.10	−0.39	−1.52	−1.43	−0.48	−1.06
ZNF367	Zinc finger protein 367	0.00	0.32	0.02	−0.57	−0.30	−0.33	−1.39	−0.76	−4.92	−5.78	−3.30	−1.19
ZNF37A	zinc finger protein 37A	0.00	−0.09	−0.18	−0.21	0.01	−0.10	−1.05	−0.55	−1.00	−0.83	−0.42	−0.52
ZNF551	Zinc finger protein 551	0.00	0.22	−0.01	−0.47	−0.16	−0.13	−1.07	−0.64	−1.66	−1.55	−0.67	−0.75
ZNF620	Zinc finger protein 620	0.00	−0.13	−0.24	−0.24	−0.10	−0.28	−1.14	−0.96	−2.06	−1.26	−0.78	−0.96
ZNF718	zinc finger protein 718	0.00	−0.11	−0.04	−0.24	−0.03	−0.14	−1.05	−0.69	−1.85	−1.42	−0.86	−1.31
ZNF729	Zinc finger protein 729	0.00	−0.11	0.05	−0.10	0.07	0.04	−1.12	−0.74	−3.69	−3.57	−2.22	−0.59
ZNF775	Zinc finger protein 775	0.00	0.08	0.18	−0.24	0.41	−0.10	0.61	1.01	1.33	1.40	1.11	0.41
ZNF782	Zinc finger protein 782	0.00	−0.14	−0.23	−0.40	−0.14	−0.28	−1.44	−0.86	−2.15	−1.60	−0.48	−1.36

*Genes were selected based on expression in 6–8 h or 18–24 h time bins. TGFβ1, the doxycycline-induced transgene, served as an internal control. Log2 ratio values are colored where red denotes increased in the mesenchymal state and green denotes decreased in the mesenchymal state*.

Genes from the 6–8 and 18–24 h sets (16,058 genes each) were used for GSEA correlations using oncogene and curated signature datasets. The top ranked gene signatures, divided into functional families and correlating with early and middle time points are shown in Table 6. The LEF1 and NFκB activation signatures and BMI1 and EZH2 inhibition signatures were correlated with the mesenchymal state at early time points. Interestingly, HOXA9 target gene inhibition also was correlated with the mesenchymal state, where crosstalk between HOXA9 and BMI1 can impact cell cycling and senescence programs (92). The positive correlation of the mesenchymal state with HIF1 hypoxia signaling, embryonic stem cell programs, and STAT signaling were observed at the early time points. A mesenchymal correlation with Myc target inhibition also was an early event (Table 6). Multiple cell cycle signatures correlated the mesenchymal state with cell cycle inhibition at early time points, consistent with previous complete EMT endpoint data (Tables 1 and 2).

**Table 6:** **Gene-set enrichment (GSEA) for RNA showing a close to or greater than twofold change between mesenchymal and epithelial cell states, by 6–8 h or by 18–24 h time bins**.

Phenotype	Time bin (h)	GSEA dataset	Size	NES	NOM *p*-value	FDR *q*-value
**TRANSCRIPTION**						
Mes	18–24	LEF1_UP.V1_UP	131	−2.13	0.000	0.000
Mes	6–8	LEF1_UP.V1_UP	131	−1.14	ns	ns
Mes	18–24	HINATA_NFKB_TARGETS_KERATINOCYTE_UP	73	−2.57	0.000	0.000
Mes	18–24	BMI1_DN.V1_UP	121	−2.25	0.000	0.000
Mes	6–8	BMI1_DN.V1_UP	121	−2.25	0.000	0.000
Mes	18–24	BMI1_DN_MEL18_DN.V1_UP	121	−2.84	0.000	0.000
Mes	6–8	BMI1_DN_MEL18_DN.V1_UP	121	−2.84	0.000	0.000
Mes	18–24	PRC2_EZH2_UP.V1_DN	146	−2.11	0.000	0.000
Mes	6–8	PRC2_EZH2_UP.V1_DN	146	−2.11	0.000	0.000
Epi	18–24	PRC2_EZH2_UP.V1_UP	145	1.74	0.000	0.003
Epi	6–8	PRC2_EZH2_UP.V1_UP	145	1.74	0.000	0.003
Epi	18–24	HOXA9_DN.V1_DN	168	1.57	0.001	0.023
Epi	6–8	HOXA9_DN.V1_DN	168	1.57	0.001	0.023
Mes	18–24	HOXA9_DN.V1_UP	163	−1.80	0.000	0.001
Mes	6–8	HOXA9_DN.V1_UP	163	−1.80	0.000	0.001
Epi	18–24	ELVIDGE_HYPOXIA_DN	142	2.31	0.000	0.000
Mes	18–24	ELVIDGE_HYPOXIA_UP	158	−2.26	0.000	0.000
Epi	18–24	MANALO_HYPOXIA_DN	285	3.24	0.000	0.000
Mes	18–24	MANALO_HYPOXIA_UP	172	−2.78	0.000	0.000
Mes	18–24	ESC_J1_UP_EARLY.V1_UP	137	−1.92	0.000	0.000
Mes	6–8	ESC_J1_UP_EARLY.V1_UP	137	−1.92	0.000	0.000
Mes	18–24	ESC_J1_UP_LATE.V1_UP	135	−2.16	0.000	0.000
Mes	6–8	ESC_J1_UP_LATE.V1_UP	135	−2.16	0.000	0.000
Epi	18–24	GARCIA_TARGETS_OF_FLI1_AND_DAX1_DN	154	2.79	0.000	0.000
Mes	18–24	GARCIA_TARGETS_OF_FLI1_AND_DAX1_UP	52	−2.22	0.000	0.000
Epi	18–24	GARY_CD5_TARGETS_DN	418	2.62	0.000	0.000
Mes	18–24	GARY_CD5_TARGETS_UP	441	−2.22	0.000	0.000
Epi	18–24	MISSIAGLIA_REGULATED_BY_METHYLATION_DN	119	2.66	0.000	0.000
Mes	18–24	MISSIAGLIA_REGULATED_BY_METHYLATION_UP	112	−2.88	0.000	0.000
Mes	18–24	WIERENGA_STAT5A_TARGETS_GROUP1	106	−2.29	0.000	0.000
Mes	18–24	WIERENGA_STAT5A_TARGETS_GROUP2	45	−2.34	0.000	0.000
Mes	18–24	WIERENGA_STAT5A_TARGETS_UP	170	−2.43	0.000	0.000
Mes	18–24	DUTERTRE_ESTRADIOL_RESPONSE_24HR_DN	482	−2.79	0.000	0.000
Epi	18–24	DUTERTRE_ESTRADIOL_RESPONSE_24HR_UP	305	3.29	0.000	0.000
Mes	18–24	MYC_UP.V1_DN	134	−2.33	0.000	0.000
Mes	6–8	MYC_UP.V1_DN	134	−2.33	0.000	0.000
Epi	18–24	MYC_UP.V1_UP	158	2.45	0.000	0.000
Epi	6–8	MYC_UP.V1_UP	158	2.45	0.000	0.000
**CELL CYCLE**						
Mes	18–24	RB_DN.V1_DN	114	−2.01	0.000	0.000
Mes	6–8	RB_DN.V1_DN	114	−2.01	0.000	0.000
Epi	18–24	RB_DN.V1_UP	125	1.54	0.008	0.027
Epi	6–8	RB_DN.V1_UP	125	1.54	0.008	0.027
Epi	18–24	RB_P107_DN.V1_UP	119	2.22	0.000	0.000
Epi	6–8	RB_P107_DN.V1_UP	119	2.22	0.000	0.000
Mes	18–24	E2F1_UP.V1_DN	164	−2.17	0.000	0.000
Mes	6–8	E2F1_UP.V1_DN	164	−2.17	0.000	0.000
Epi	18–24	E2F1_UP.V1_UP	171	1.87	0.000	0.001
Epi	6–8	E2F1_UP.V1_UP	171	1.87	0.000	0.001
Mes	18–24	CHANG_CORE_SERUM_RESPONSE_DN	194	−3.22	0.000	0.000
Epi	18–24	CHANG_CORE_SERUM_RESPONSE_UP	208	2.26	0.000	0.000
Mes	18–24	CSR_LATE_UP.V1_DN	136	−2.84	0.000	0.000
Mes	6–8	CSR_LATE_UP.V1_DN	136	−2.84	0.000	0.000
Epi	18–24	CSR_LATE_UP.V1_UP	149	2.36	0.000	0.000
Epi	6–8	CSR_LATE_UP.V1_UP	149	2.36	0.000	0.000
Epi	18–24	MOLENAAR_TARGETS_OF_CCND1_AND_CDK4_DN	56	2.60	0.000	0.000
Mes	18–24	MOLENAAR_TARGETS_OF_CCND1_AND_CDK4_UP	56	−2.23	0.000	0.000

*Signatures enriching from transcriptional regulation (top panel) and cell cycle regulation (bottom panel) are shown. Individual GSEA signatures are defined in detail at http://www.broadinstitute.org/gsea/msigdb/index.jsp*.

## Discussion

A detailed investigation of molecular differences contrasting isogenic epithelial and stem-like mesenchymal tumor cell states has been undertaken in isogenic lung adenocarcinoma cell models harboring mutant-KRas. Our observations reinforce the important role that cancer stemness and EMT can have in driving drug resistance in tumor cells (7, 8, 12, 17, 93) and highlight the wide diversity of mechanisms (94) that can be used by tumor cells to evade targeted- and chemo-therapies. Agents and combinations successfully targeting mutant-KRas containing tumor cells, in an epithelial or mesenchymal cell context, would have a marked impact in the treatment of NSCLC and particularly pancreas cancer where trans-differentiation is a frequent event (22). An understanding of these mechanisms and molecular contexts will have important implications in driving combinatorial drug therapy in cancer patients in the future.

Mutation of the KRas oncoprotein leads to the interaction and activation of BRaf/CRaf and Mek1/2 kinases and phosphorylation of Erk1/2 on the activation segment. Erk1/2 phosphorylation on the loop TXY motif activated Erk1/2 catalytic activity and leads to downstream cytoplasmic and nuclear pro-survival substrate target phosphorylation. In both A549 and H358 models, a markedly increased Erk active site phosphorylation was observed in the mesenchymal state (Table S3 in Supplementary Material). Similar to the H358 and A549 models described here we have observed TGFβ mediated induction of EMT in mt-EGFR NSCLC adenocarcinoma lines HCC4006 and HCC827 (data not shown). A common feature in both mt-KRas and mt-EGFR models is an elevated activation of the Erk1/2 kinases. These data support a hypothesis that chronic Erk activation may contribute to the initiation of EMT, and once EMT has occurred, Erk is further activated in part through integrin/paxillin/FAK and TGFbetaR1 signaling networks. Thus we suggest that KRas, through Erk, may act as both to prime initiation of EMT and to maintain the mesenchymal state once a transition has occurred. Preliminary RNA interference studies indicate enhanced mesenchymal cell sensitivity to Erk1/2 knockdown. Interestingly, synthetic lethality in multiple mt-KRas backgrounds has been observed with knockdown of Snail2/Slug (95).

Though our principle interest is in identifying key survival targets required for the viability of cells in the mesenchymal state, we considered that druggable transcriptional or epigenetic regulators acting early in the EMT process might serve as useful candidates. Previous studies have highlighted the heterogeneity of distinct EMT programs and the plethora of potential targets, for example, FGFR (57), Axl (96), PDGFR (97), JAK-STAT (98, 99), FAK (100), contributing to the viability of mesenchymal-like tumor cells in a model specific manner. Given the complex nature of epigenetic changes occurring as cells transition from epithelial to mesenchymal states, we specifically defined networks, which can modulate epigenetic states. The rationale is that pharmacological modulation of epigenetic regulators can alter the expression of many potential mesenchymal drug targets, potentially overcoming the redundancy and heterogeneity of mesenchymal therapeutic targets. Here, we have identified multiple epigenetic regulatory networks, for example, the PRC1 complex, HP1γ, and BAF/Swi-Snf complex, which can contribute to the formation and maintenance of epithelial and mesenchymal tumor cell states. Overlapping transcriptional programs also were observed, for example, the redundant loss of epithelial state transcription (GRHL2, TRAPC2, EHF, and OSR2) and splicing (ESRP1 and ESRP2) factors. This theme of modulation of overlapping or redundant network components was also observed for enhancers of the mesenchymal cell state (e.g., overlapping Zeb1/2, Snail1/2, Twist) and chromatin reprograming machinery. Synthetic lethality and therapeutic reprograming of the mt-KRas NSCLC models can be investigated by future RNAi or CRISPR approaches and/or cDNA overexpression studies, using the transcriptional and epigenetic nodes defined.

## Conflict of Interest Statement

The authors declare that the research was conducted in the absence of any commercial or financial relationships that could be construed as a potential conflict of interest.

## Supplementary Material

The Supplementary Material for this article can be found online at http://www.frontiersin.org/Journal/10.3389/fonc.2014.00344/abstract

Table S1 **Excel format table of RSEM normalized H358 and A549 genes from RNA-Seq, edited where any read is non-zero**.

Table S2 **Excel file of H358 and A549 phosphopeptides identified at 95% confidence from ProteinPilot v4.0, where individual peptide spectra were acquired four or more times**.

Table S3 **Excel file of concordant phosphopeptide changes in the H358 and A549 models (where *N* ≥ 4)**.

Table S4 **Concordant protein changes between A549 and H358 models. All proteins were identified at >95% confidence with two or more unique peptides to conform to recommendations of Bradshaw et al. (**38). Protein isoform abundance was measured by LC-MS/MS of SILAC labeled peptides and ratios between mesenchymal and epithelial cell states log2 linear scaled. Log2 ratio values in italics reflect *p* < 0.05. The number of unique peptides identified at 95% confidence, not shared with related proteins, are shown.

Table S5 **Expected benchmark GSEA and pathway prediction signatures (IPA) for stemness, TGFβ signaling, and mutant-KRas signaling in H358 and A549 KRas NSCLC models comparing isogenic epithelial and mesenchymal cell states**. These correlations serve as expected benchmarks for **(A)** stemness (Mani, Thomson ref), active TGFβ signaling (EMT inducer in both models) and **(B)** KRas signaling (KRas oncogene in both models). KRas signatures were enhanced in the mesenchymal cell state relative to the isogenic epithelial state, consistent with enhanced phospho-Erk in mesenchymal A549 and H358 cell states (23, 57). Individual GSEA signatures are defined in detail at http://www.broadinstitute.org/gsea/msigdb/index.jsp.

Table S6 **Reference RNA expression of transcriptional inducers of EMT (top panel) and potential markers of stemness (bottom panel)**.

Table S7 **GSEA and pathway prediction analysis (IPA) shows statistically significant enrichment of BMI1 target genes altered with EMT state**. Manual analysis of BMI1 target RNA expression indicating divergence in the direction of signaling between H358 and A549 and divergence within each model, suggesting while BMI1 target genes are enriched and differentially expressed with changing EMT state, the direction is not uniform and other factor(s) likely contribute along with BMI1.

Figure S1 **Immunoblot staining for fibronectin, E-cadherin, vimentin, the doxycycline-induced TGFβ1 transgene, and actin (control) in the H358-dox-TGFβ model in epithelial (−dox) or mesenchymal (+dox) states (5 days on doxycycline)**.

## Footnotes

^1^http://www.expressionanalysis.com

^2^http://david.abcc.ncifcrf.gov/
