# Supplementary figures and images for: Altered Transcriptional Control Networks with Trans-Differentiation of Isogenic Mutant-KRas NSCLC Models
Source: Front Oncol. 2014 Dec 8;4:344. doi: 10.3389/fonc.2014.00344 (PMC4259114; doi:10.3389/fonc.2014.00344)

### H358/doxTGF $\beta$

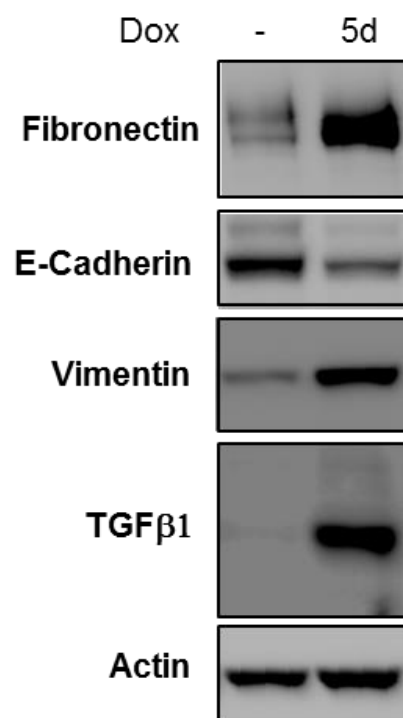

Supplement: Table S1 — Excel format table of RSEM normalized H358 and A549 genes from RNA-Seq, edited where any read is non-zero. [file Data_Sheet_1.ZIP › FigureS1.pdf]
